# Lived Experiences of Social Isolation and Meaningful Relationships Among Older Adults Living with HIV with a Concurrent Mental Health Diagnosis: A Heideggerian Phenomenological Approach

**DOI:** 10.3390/healthcare14020257

**Published:** 2026-01-20

**Authors:** Kristina M. Kokorelias, Dean Valentine, Andrew D. Eaton, Sarah E. P. Munce, Christine L. Sheppard, Sander L. Hitzig, Marina B. Wasilewski, Alice Zhabokritsky, Reham Abdelhalim, Laura Jamieson, Maurita T. Harris, Luxey Sirisegaram

**Affiliations:** 1 Department of Medicine, Healthy Ageing and Geriatrics Program, Sinai Health, University Health Network, 600 University Ave, Toronto, ON M5G 1X5, Canada; 2 Department of Occupational Science and Occupational Therapy, Temerty Faculty of Medicine, University of Toronto, 500 University Avenue, Toronto, ON M5G 1V7, Canada; 3 Rehabilitation Sciences Institute, Temerty Faculty of Medicine, University of Toronto, 500 University Avenue, Toronto, ON M5G 1V7, Canada; 4 Jane Addams College of Social Work, University of Illinois at Chicago, 1040 W. Harrison St. 4th Floor, Chicago, IL 60607, USA; 5 Institute of Health Policy, Management and Evaluation, University of Toronto, Toronto, ON M5G 1V7, Canada; 6 Bloorview Research Institute, Holland Bloorview Kids Rehabilitation Hospital, Toronto, ON M4G 1R8, Canada; 7 Factor-Inwentash Faculty of Social Work, University of Toronto, Toronto, ON M5S 1V4, Canada; 8 St. John’s Rehab Research Program, Sunnybrook Research Institute, Sunnybrook Health Sciences Centre, Toronto, ON M2M 2G1, Canada; 9 Dalla Lana School of Public Health, University of Toronto, Toronto, ON M5T 3M7, Canada; 10 Department of Medicine, University of Toronto, Toronto, ON M5S 3H2, Canada; 11 Division of Infectious Diseases, University Health Network, Toronto, ON M5G 2C4, Canada; 12 Burlington Ontario Health Team, Burlington, ON M5G 1X5, Canada; 13 Faculty of Liberal Arts, Wilfrid Laurier University, Brantford, ON N3T 2Y3, Canada

**Keywords:** HIV, older adults, mental health, social isolation, loneliness, meaningful relationships, phenomenology, Social Convoy Theory, peer support, virtual engagement

## Abstract

**Highlights:**

**What are the main findings?**

- Older adults living with HIV and a concurrent mental health diagnosis experience social isolation as both a physical and existential state, shaped by stigma, mental health challenges, and the spatial and cultural contexts of their lives.
- Meaningful relationships are defined by authenticity, trust, emotional safety, and reciprocity, often occurring within peer networks of individuals with shared HIV or mental health experiences and facilitated by both community engagement and virtual platforms.

**What are the implications of the main findings?**

- Interventions to reduce social isolation should focus not only on increasing social contact but on creating affirming, context-sensitive spaces that support disclosure, trust, and reciprocal relationships.
- Policies and programs targeting older adults with HIV and mental health challenges must consider emotional and identity-related dimensions of social connection, emphasizing validation, agency, and continuity of relational experiences.

**Abstract:**

**Background/Objectives:** Meaningful social connections are critical for well-being in later life, yet older adults living with HIV frequently experience social isolation and loneliness, compounded by stigma, mental health conditions, and systemic inequities. This study aimed to explore how older adults living with HIV and a concurrent mental health diagnosis experience social isolation and cultivate meaningful relationships, situating these experiences within Social Convoy Theory. **Methods:** Using a Heideggerian phenomenological approach, we conducted in-depth, semi-structured interviews with 33 adults aged 50 and older in Ontario, Canada, who self-identified as living with HIV and a diagnosed mental health condition. Participants were recruited through community-engaged strategies and snowball sampling. Data were analyzed iteratively, combining descriptive and interpretive coding to identify patterns in social isolation, relational meaning, and the influence of intersecting social, structural, and health determinants. **Results:** Participants described social isolation as both a physical and existential experience, influenced by stigma, mental health challenges, and contextual factors such as urban versus rural settings. Meaningful relationships were characterized by authenticity, trust, emotional safety, and reciprocity, often formed within peer networks sharing similar lived experiences. Community engagement and virtual platforms facilitated connection, while rural or suburban environments often intensified isolation. Relationships providing validation, agency, and continuity of experience were particularly impactful on participants’ well-being. **Conclusions:** Social isolation among older adults living with HIV and mental health conditions extends beyond objective network measures to include emotional and identity-related dimensions. Interventions should prioritize affirming, context-sensitive spaces that support disclosure, trust, and reciprocal relationships, recognizing the nuanced needs of this population for both social and existential connectedness.

## 1. Introduction

Meaningful social connections are a cornerstone of well-being in later life [1], yet many older adults experience social isolation, loneliness, and disruptions in their support networks [2]. These experiences can have profound effects on mental and physical health (e.g., early onset of chronic diseases, depression, etc.), influencing resilience, quality of life, and the ability to navigate healthcare systems [2]. For older adults living with HIV, social relationships are often further complicated by stigma, discrimination, and the intersection of multiple health and social vulnerabilities, including mental health concerns, age-related changes, and systemic inequities [3,4]. A qualitative study in Ottawa, Canada identified three dominant themes regarding mental health among older adults living with HIV (age 52–67): uncertainty (about survival, symptoms and future), stigma (health care, appearance, compounded by HIV and age), and resilience strategies (including social support engagement) [5].

Ageing in the context of HIV is also accompanied by transitions such as retirement, losses of peers or partners, shrinking social networks, changes in role and identity [6]. While these experiences are true for many older adults, for those with living HIV, additional layers of complexity exist (e.g., earlier peer losses during the HIV epidemic, disclosure concerns, intersection with sexual orientation or gender identity) [6]. Using practical coping strategies, such as seeking social support and focusing on solutions, is linked to reduced levels of depression, social isolation and loneliness [7]. However, older adults living with HIV face high rates of loneliness and social isolation [8]. We define social isolation as the *objective* state of having minimal social contacts, infrequent interactions and limited network size, often measured by membership in community groups, volunteering status, or living arrangements [9]. However, loneliness is the *subjective* emotional experience arising from a perceived gap between one’s desired and actual social relationships, whether in quantity or quality [10]. Loneliness and social isolation among older adults with HIV have been linked to poorer self-management, negative health behaviors, and adverse physiological and psychological outcomes [8,11]. Social isolation among older adults living with HIV has been associated with higher rates of hospitalization and mortality than their non-HIV positive peers [6].

Meaningful relationships are a nuanced construct, often referring to social ties that provide emotional support, affirmation of identity or belonging, trust and relational continuity, and contribute to one’s sense of purpose or connectedness [12]. Many studies are cross-sectional, longitudinal or mixed-methods designs that explore how relationships evolve, how isolation is experienced (subjectively and objectively), and how mental health diagnoses shape these dynamics are sparse [8]. However, the intersection of HIV, ageing and mental health must be considered when exploring social and relational experiences [13,14]. Older adults living with HIV who also carry a concurrent mental health diagnosis face compounding vulnerabilities, as mental health conditions may impede social engagement (through withdrawal, low mood, cognitive impairment, stigma) [15], and social isolation may in turn exacerbate mental health conditions [16], creating a bidirectional relationship and stigma.

Social Convoy Theory offers a valuable framework for understanding how individuals’ social networks evolve and function across the life course [17]. The theory conceptualizes social relationships as a “convoy”, that is defined as a network of significant others who provide emotional, informational, and practical support as people navigate different stages and circumstances of life [17]. These convoys are dynamic, shaped by both personal characteristics (such as health, gender, and personality) and contextual factors (such as culture, socioeconomic position, and major life transitions) [17]. Over time, the composition, quality, and perceived stability of one’s convoy can shift in response to changes in physical health, cognitive functioning, social roles, or life losses [17]. In later life, convoys often become smaller but more emotionally meaningful, reflecting a natural narrowing of social engagement toward relationships that provide the greatest sense of satisfaction and support [17]. For older adults living with HIV, however, these networks may be more fragmented or fluid [18]. Social Convoy Theory extends beyond simple measures of network size or contact frequency to highlight how individuals experience the *quality* of social ties, such as trust, reciprocity, emotional closeness, and how these contribute to well-being [18,19].

This study aims to explore the lived experiences of social isolation and meaningful relationships among older adults living with HIV a concurrent mental health diagnosis, with particular attention to how intersecting biological, social, and structural determinants shape these experiences. This study positions Social Convoy Theory as a guiding framework to interpret how older adults living with HIV and concurrent mental health diagnoses experience social isolation and meaningful relationships. Concurrent mental health diagnosis refers to the presence of a clinically recognized mental health condition in addition to living with HIV. Phenomenology offers a rigorous approach for capturing the essence of lived experiences, allowing researchers to understand how social isolation and relationships are experienced and interpreted by older adults living with HIV. By centering participants’ voices, phenomenology provides insight into the meanings they assign to social connections, the strategies they use to maintain relationships, and how structural and social determinants influence these experiences [20].

## 2. Methodology

This study adopts a Heideggerian phenomenological approach to explore the lived experiences of older adults living with HIV who also have a diagnosed mental health condition [21]. Phenomenology, as both a philosophical stance and a methodological approach, is concerned with understanding human experience from the perspective of those who live it [21]. In taking up Heidegger’s articulation, we focus on the meaning and essence of social isolation and relationships as experienced by participants, acknowledging that consciousness and perception are inseparable from the social, cultural, and structural contexts in which people live [21].

Older adults living with HIV occupy a unique social and embodied space. Heideggerian phenomenology allows us to ask, *“What is it like to experience social isolation and meaningful relationships while ageing while living with HIV and a mental health diagnosis?”.* The use of Heideggerian phenomenology draws attention to intentionality, the ways in which participants orient themselves toward others, relationships, and their social worlds, as well as the substance of consciousness as it is lived in everyday contexts [22]. Unlike Husserlian phenomenology, Heideggerian phenomenology does not assume that experiences can be studied in isolation from the world [22]. Instead, it emphasizes being-in-the-world, recognizing that individuals’ experiences of social connection and isolation are inseparable from their histories, environments, and interactions with others [22]. In this study, we explore participants’ lived experiences of social isolation, the processes through which meaningful relationships are cultivated and sustained, and the role of intersecting determinants such as mental health, HIV-related stigma, ageing, and socio-structural contexts. We intentionally situate the study in participants’ everyday worlds to understand how they make sense of social connection and disconnection in ways that are meaningful to them. By combining Heideggerian phenomenology with a focus on social convoy theory, this methodology allows for a nuanced exploration of both the structure and meaning of social relationships for older adults living with HIV and a concurrent mental health diagnosis.

While Social Convoy Theory encompasses protective factors at multiple levels, in this study we operationalized the theory to focus specifically on participants’ perceptions of their social ties and the meaning they ascribe to relationships. Interviews and analysis emphasized relational quality, reciprocity, and emotional support, rather than structural metrics such as network size or social capital. This approach allowed us to capture the lived experience of social connection and isolation in context, consistent with our phenomenological framework.

This study was approved by the Sinai Health Research Ethics Board (REB: 23-0106-E).

### 2.1. Recruitment

Eligible participants were adults aged 50 years or older who self-identified as living with HIV and a diagnosed mental health condition, were able to communicate in English, had experience accessing mental health services in the Greater Toronto Area, Ontario or Ottawa, ON, Canada, and could provide informed consent. Exclusion criteria included those experiencing acute psychiatric instability or cognitive impairment that would interfere with participation, or those who were experiencing a current hospitalization or crisis. In keeping with the phenomenological focus on lived experience, participants were recruited using community-engaged strategies designed to reach individuals embedded in their social worlds. Recruitment included outreach at community events, distribution of flyers in culturally diverse venues, and announcements through AIDS Service Organization (ASO) websites and social media platforms. Additionally, snowball sampling was employed to allow participants to connect the research team with peers who could offer rich experiential insights [23].

Potential participants-initiated contact with a research coordinator and completed a brief eligibility screening, including a short demographic survey capturing characteristics such as sex and geographic location. Maximum variation sampling was then applied to ensure a diverse range of experiences and perspectives, consistent with phenomenology’s aim of capturing the breadth and depth of lived experience [24]. Recruitment continued iteratively until data saturation was achieved, reflecting the point at which no new experiential themes were emerging [25]. To determine saturation, we conducted iterative, concurrent data collection and analysis. After each set of interviews, we reviewed the transcripts to identify emerging themes and monitored whether new interviews were providing novel insights. Saturation was considered reached when no new themes, categories, or significant variations in experiences were observed across consecutive interviews, indicating that the data sufficiently captured the range of participant perspectives.

In total, 33 participants were included. While phenomenological studies often use small samples to focus on the richness of individual experiences, sample sizes are flexible and should reflect the scope and complexity of the research question [24]. Methodological reviews suggest that phenomenological studies frequently include between 4 and 25 participants, with larger samples sometimes used to capture greater diversity [24]. The additional participants allowed us to maximize variation across factors such as gender, ethnicity, geographic location, and mental health service use, enhancing the credibility and transferability of the findings while still maintaining a manageable dataset for in-depth phenomenological analysis. This approach aligns with contemporary recommendations emphasizing that phenomenological sample sizes should be sufficient to answer the research question and capture the breadth of lived experiences, rather than adhering strictly to a fixed number [24].

### 2.2. Data Collection

Data were collected through in-depth, semi-structured interviews, conducted as part of a broader program of research examining HIV and mental health system navigation among older adults living with HIV. No pilot interviews were conducted. Prior to the interviews participants were given the option to complete an optional demographic form to contextualize their responses. To create a safe and respectful space for participants, we chose not to ask for specific mental health or HIV diagnoses. This decision was made to prioritize participants’ comfort, reduce potential distress, and emphasize the study’s focus on lived experience and relational meaning rather than medical labels.

During the interviews, topics such as social networks, support systems, and relationship dynamics arose organically in participants’ narratives. In subsequent interviews (from the third interview onward), these topics were intentionally probed with the remaining participants to ensure that data captured the full depth and breadth of participants’ social experiences. Interviews, therefore, often focused on participants’ experiences of social connection and disconnection, the processes through which they cultivate and maintain meaningful relationships, and the impact of intersecting factors such as mental health, stigma, ageing, and HIV-related health challenges. Participants were also invited to reflect on their experiences navigating mental health services in Ontario, situating relational experiences within broader social and structural contexts. Open-ended questions were designed to elicit narratives rather than brief responses, allowing participants to describe events, emotions, intentions, and relational dynamics in their own words. Probing questions were used to encourage participants to expand on significant experiences, relationships, or moments of isolation that shaped their perceptions of connection and well-being.

Interviews were conducted either in person (*n* = 2) or virtually (*n* = 31), depending on participant preference and accessibility, and lasted approximately 60 to 90 min. Interviews were conducted by a Masters-level trained female research coordinator or a male individual with lived experience of HIV and a mental health diagnosis. Participants could choose who they wanted to be interviewed by. Both reviewers kept a reflexive journal throughout the interview process and had frequent conversations with the research team about their own positionality [26]. The goal was to co-construct meaning with participants by creating a conversational, respectful space in which they could articulate the essence of their experiences with social isolation, relational support, and mental health navigation. All interviews were audio-recorded with participant consent and transcribed verbatim by a transcriptionist to preserve the richness of participants’ accounts. To protect anonymity, participants were assigned pseudonyms for each transcript and other identifying details were removed (e.g., workplaces, physician names). Field notes were taken during and immediately after interviews to capture contextual information, non-verbal cues, and researcher reflections, which were incorporated into subsequent interpretive analysis. All participants received a gift card honorarium for their time.

### 2.3. Data Analysis

Data were analyzed using a Heideggerian phenomenological approach, which emphasizes interpretation (hermeneutics) and seeks to understand the essence of lived experience in context [27]. The analytic process began with the first and senior author, as well as the researcher with lived experience and a research assistant reading and re-reading of transcripts, to identify preliminary patterns and notable experiential descriptions. This stage focused on the ontic level (i.e., factual, descriptive, observable details), attending to participants’ descriptions of daily life, social interactions, and relational dynamics, without imposing pre-existing assumptions or theoretical categories [28]. Following this, transcripts were coded iteratively by all those individuals, attending both to descriptive elements (e.g., experiences of isolation, social support structures, coping strategies) and interpretive elements (e.g., how participants assign meaning to relationships, the impact of stigma, or the role of mental health in shaping social engagement). Coding aimed to emphasize participants’ own language, intentions, and contextualized experiences rather than imposing preconceived categories [21,29]. A sample of the codebook is found in Table 1. First-level coding emphasized descriptive, factual elements of participants’ narratives, including daily activities, social interactions, and concrete experiences of isolation or support. However, following the first round of coding, open-ended codes were mapped onto convoy-related concepts, including the composition of social ties, perceived closeness, reciprocity, and the support functions provided by different network members. During this phase, research team discussions aimed to uncover phenomena hidden within participants’ narratives. This required interpreting the meanings behind participants’ experiences of social isolation, relationship maintenance, and social support, reflecting on how participants make sense of their being-in-the-world when confronted with HIV, mental health challenges, and ageing [30]. Thus, second-level coding involved interpretive analysis, identifying patterns, meanings, and relational dynamics, and mapping these onto Social Convoy Theory concepts. This two-stage process was iterative and reflexive, with team discussions and journaling used to challenge assumptions and ensure interpretations remained grounded in participants’ lived experiences.

From there, the analysis process was iterative and reflexive. Research team members engaged in regular analytic discussions, reviewing coded transcripts and preliminary themes to interrogate interpretations, challenge assumptions, and ensure alignment with phenomenological principles. Reflexive journaling captured researchers’ perspectives and potential biases, acknowledging that interpretation is inevitably influenced by the researchers’ own experiences while striving to remain grounded in participants’ narratives.

During discussions, transcripts were returned to such that the research team could determine convergence and divergence in experiences amongst the participants, and highlighting the ways that social isolation, meaningful relationships, and system navigation intersected with participants’ identities, histories, and embodied realities.

Credibility was enhanced through investigator triangulation, reflexive journaling, and iterative team-based interpretation. Emerging interpretations were discussed within the research team, including a researcher with lived experience, to ensure resonance with participants’ accounts. The use of thick description and representative quotations supports transparency and allows readers to assess the trustworthiness and transferability of the analysis.

## 3. Results

A total of 33 individuals participated in the study, with thirty providing complete demographic information. Participants’ ages spanned from 50 to over 80 years, with the largest group falling between 55 and 64 years. Most resided in urban settings. Twenty-one participants indicated English as their primary language, while nine reported speaking another language. Nineteen identified as men, 10 as women, and one as non-binary. A majority reported annual household incomes under $30,000 and lived alone. Several participants described living with multiple physical disabilities, including visual, hearing, and mobility impairments, while twelve participants reported no disabilities. Key characteristics of the sample are summarized in Table 2.

Participants’ narratives revealed the complex, deeply relational nature of ageing with HIV and a concurrent mental health diagnosis. The themes that emerged from participants’ experiences are ‘Community as Lifeline and Antidote to Isolation’, ‘Peer Mentorship and Reciprocity’, ‘Accessibility and Virtual Connection as a Bridge and Social Worlds as Lived Context’. These themes describe how participants define and maintain connections through community, trust, and virtual spaces. Experiences of social isolation and meaningful connection were fluid, influenced by changes in health, stigma, and access to supportive spaces. Meaningful relationships were defined more by authenticity, mutual understanding, and emotional safety than by frequency of contact, often found with peers who shared similar lived experiences, regardless of age or mentoring roles. While many described deep loneliness and the loss of social ties due to illness, stigma, or grief, they also expressed a strong desire for belonging and emotional reciprocity. Participants emphasized that social isolation was not only physical but existential, rooted in feeling “unseen or misunderstood.”

Each theme reflects aspects of participants’ social convoys, including the composition of their networks, the quality and closeness of relationships, the reciprocity and support functions provided, and the ways in which ties are maintained or adapted over time. Themes such as community as a lifeline, peer mentorship, virtual connection, and social worlds as lived context demonstrate how convoy structure and relational meaning jointly shape experiences of social isolation, connection, and emotional well-being. Sub-themes related to stigma, selective disclosure, and accessibility further illustrate how participants navigate and negotiate their social networks in ways that preserve trust, authenticity, and emotional safety, highlighting the dynamic and context-dependent nature of social convoys for older adults living with HIV and concurrent mental health conditions.

Participants’ experiences of social connection and isolation varied across demographic and contextual factors. Older participants, particularly those over 70, often described more limited peer networks and greater reliance on virtual or structured community programs, whereas younger participants engaged more actively in mentorship and advocacy roles. Urban residents generally had greater access to peer networks and support services compared with those in suburban or rural areas, who reported heightened isolation. Experiences also differed by gender and mental health status: men more frequently described peer mentorship roles, while participants managing anxiety or depression highlighted the importance of emotionally safe spaces for disclosure. These variations underscore how intersecting social, geographic, and health-related factors shape ageing with HIV and concurrent mental health challenges.

We describe each theme below with quotes from participants. We source each anonymized quote by participant ID, sex and age.

### 3.1. Theme 1: Community as Lifeline and Antidote to Isolation

Participants consistently described community as central to their survival and well-being, particularly when navigating HIV and mental health challenges. Meaningful relationships were often characterized as mutual, supportive, and non-judgmental, often involving peers who share lived experience with HIV, mental health, or substance use. As one participant reflected, “*the opposite of addiction is community*.” (Participant 3, Male, 62). Feeling acknowledged and valued by healthcare providers also contributed to this sense of community, as interactions that convey respect and trust, such as being called by one’s first name or receiving gentle, caring touch, enhanced relational intimacy. Beyond healthcare, safe housing environments with peers living with HIV provide critical social support: “*I live in a Co-OP that has a very high percentage of people with HIV that are safe in their subsidized housing, and I wish there were more. Being around them knowing they understand me and HIV is what is my lifeline when I want to hide away*” (Participant 5, Male, 67).

### 3.2. Theme 2: Peer Mentorship and Reciprocity

Participants described finding meaning in relationships that allowed them to give back through mentoring, advocacy, or facilitating peer support. They emphasized that meaningful relationships are reciprocal, involving the exchange of knowledge, lived experience, and support. For many, aging while living with HIV and navigating mental health challenges, particularly anxiety and depression, prompted reflection on life experiences, and relationships felt most meaningful when they validated these experiences while offering opportunities for agency and contribution. Many older adults derived purpose from mentoring newly diagnosed individuals, regardless of age. One participant reflected on mentoring younger men living with HIV. “*Sharing decades of my experience helps them navigate stigma and health challenges. It feels meaningful because I can guide them in ways I wish I had been guided*.” (Participant 14, Male, 56). Community centers and social groups provided spaces to meet people “like them,” gain mentorship, and engage in advocacy or volunteer work that reinforced a sense of purpose.

Some participants co-facilitated formal peer groups or shared practical strategies for navigating healthcare, medications, and stigma with other older adults living with HIV. Those providing peer mentorship in formal programs offered through Aids Service Organizations. described helping others manage the trauma of diagnosis and understand complex treatment regimens, particularly for those diagnosed with a mental health condition later in life. Mentors noted that these acts reinforced their own sense of competence and belonging: “*I really try to help others navigate what I went through… so they don’t feel so lost or scared at the start because I got that mentorship*.” (Participant 7, Female, 55).

Reciprocity also extended to practical health guidance and advocacy, such as helping peers access services like the Ontario Disability Support Program (ODSP; the province’s income support program) or navigating third-party payers including private insurance for necessary medications. Participants emphasized that these exchanges require trust and nonjudgmental spaces, noting that discussions about aging, mental health, and HIV-related challenges are often rushed or overlooked in standard clinical encounters.

### 3.3. Sub-Theme: Stigma, Confidentiality, and Selective Disclosure

Participants noted that peer connections allowed them to share sensitive information without fear of negative consequences. In contrast, the inability to freely disclose personal health information to peers contributed to them being socially isolated and made it harder to form and maintain meaningful relationships. One participant emphasized the importance of confidentiality, reflecting on societal stigma: “*Many people are also uncomfortable about the HIV status being disclosed, because they don’t want anyone else to know, because of the stigma. So they often will go a while without telling anyone or they will try to not make friends with people so they don’t have to disclose. I’ve learned to be confident…but only if I need to share*” (Participant 13, Male, 65).

### 3.4. Sub-Theme: Accessibility and Virtual Connection as a Bridge

Participants highlighted the potential of virtual spaces to maintain and build social connections, especially when attending in-person activities was difficult due to mobility limitations, rural location, or stigma. Those participating in virtual mental health support groups described being able to choose whether to share their HIV status or mental health experiences, and whether to use their real names. This flexibility supported comfort and safety while fostering meaningful interactions. Participants noted that while this approach “guarded some of their identity,” it also allowed them to behave in authentic ways, such as openly discussing their experiences with medication side effects, relationship challenges, or sexual health.

Some participants used technology to connect with peers living with HIV through FaceTime, email, or messaging. Virtual relationships provided emotional support and shared understanding, often connecting participants across cities or countries. While many acknowledged that the COVID-19 pandemic accelerated virtual engagement, some had used email and online communication to maintain connections even before the pandemic. One participant described the importance of choice, saying, “*I would love to be able to go online and chat with someone and not be lonely rather than waiting until we can meet in person, rather than something that is your only choice… it really has to be a big, big voluntary component to go see your friends and sometimes a call is easiest*.” (Participant 14, Male, 78)

### 3.5. Theme 4: Social Worlds as Lived Context

Although participants valued connections with peers living with HIV or trusted friends, most described feelings of isolation that were closely linked to the social, cultural, and physical contexts where they lived. Isolation was not only physical but also emotional, rooted in feeling unseen, misunderstood, or judged. Participants in urban areas often found it easier to form friendships, join peer networks, and access support groups. In contrast, those in suburban, small-town, or rural settings experienced greater isolation, with fewer peers and resources available, which intensified feelings of being marginalized. One participant reflected, “*I sometimes feel like I’m the only one living with HIV in this town*.” (Participant 10, Female, 58)

Most participants were diagnosed with HIV before addressing mental health concerns. While relationships with peers living with HIV provided meaning and support, participants often felt isolated from people without HIV. Friends and family were generally more accepting of mental health challenges, whereas HIV continued to carry stigma and misunderstanding from community-members. For some participants, others assumed that their HIV status automatically implied they had mental health challenges, such as depression or anxiety. This conflation made it harder for them to disclose their status or seek support, because revealing one aspect of their identity risked exposing assumptions about the other. For example, one participant shared that when they told a new healthcare provider about their HIV, the provider immediately asked about therapy or psychiatric care, making them feel their physical health needs were overshadowed by assumptions about their mental health.

Even when participants had strong peer relationships with others living with HIV, they often felt a subtle divide from those peers who did not experience mental health challenges. One participant explained that while they could openly discuss medication routines or navigating stigma with peers, conversations about coping with anxiety or depression sometimes made them feel isolated, as these issues were not shared by all peers. Another described attending support groups where discussions focused on HIV management, leaving little space to explore the emotional and psychological burdens they carried, reinforcing a sense of separation.

## 4. Discussion

In this study, we used a Heideggerian phenomenological approach [27] to explore the lived experiences of social isolation and meaningful relationships among older adults living with HIV who also have a diagnosed mental health condition, situating participants’ narratives within Social Convoy Theory to understand both the structure and meaning of their social ties. We recruited 33 adults aged 50 and older through community-engaged strategies in Ontario, ensuring diversity in gender, ethnicity, geographic location, and experiences of social and mental health support. Through in-depth, semi-structured interviews, participants described social isolation as both a physical and existential experience, shaped by stigma, mental health challenges, and the spatial and cultural contexts of their lives. Meaningful relationships were characterized by authenticity, mutual understanding, emotional safety, and reciprocity, often occurring within peer networks of individuals with shared HIV or mental health experiences. Access to urban resources and virtual platforms facilitated connection, whereas rural or suburban settings often intensified isolation. Findings highlight the complex interplay between social environments, health conditions, and relational experiences, suggesting that interventions to reduce isolation and enhance well-being for this population should focus not only on increasing social contacts but on creating affirming, context-sensitive spaces that support disclosure, trust, and reciprocal relationships. Participants in our study described social isolation not only as physical separation from others, but as an existential state of feeling unseen, misunderstood, or judged by those who were either unaware of or did not share their experiences of living with both HIV and a mental health condition. This perspective extends understanding beyond traditional measures of loneliness or network size, emphasizing the emotional and identity-related dimensions of isolation among older adults living with HIV.

Our findings extend Social Convoy Theory by demonstrating that the structure and function of social networks are inseparable from the lived experiences of ageing with HIV and concurrent mental health conditions. While the theory emphasizes the composition, closeness, and support functions of social ties, our study highlights how these elements are dynamically shaped by stigma, disclosure concerns, geographic context, and health status. Specifically, participants’ reliance on reciprocal, emotionally safe relationships and selective engagement with peers or virtual networks underscores that the quality and perceived authenticity of ties may be as critical as their presence or size. These insights suggest that social convoys are not static, but adapt in response to intersecting vulnerabilities, offering a more nuanced understanding of how relational meaning and network structure jointly contribute to resilience, well-being, and coping among older adults living with HIV.

Relational meaning was not only about companionship but also about validation, agency, and continuity of experience, providing both emotional and existential support. This contrasts with some existing work suggesting that social support broadly improves health outcomes [31,32], by showing that support is most impactful when it allows for trust, validation, and identity affirmation within communities. Our results also highlight a nuanced interplay between stigma, disclosure, and social connection that has been less emphasized in previous literature [8]. These findings align in part with recent research that explored the transformative potential of disclosure in dismantling HIV-related stigma and fostering personal growth and healing [33]. In that study that was focused on WHO and what type of study design, disclosure was understood as a catalyst for empowerment and social reintegration, with participants reporting enhanced self-esteem, increased social support, and renewed purpose following open acknowledgment of their status [33]. However, that study also illuminated the risks of rejection and discrimination following disclosure, underscoring the complex, culturally contingent nature of revealing one’s HIV status [33]. Our findings echo these complexities but also noted participants’ assumptions and invisibility when having a lack of peers living with HIV rather than overt exclusion. For our participants, disclosure was often selective and deeply relational. Whereas 20 adult participants in a previous study conducted in the Democratic Republic of Congo viewed disclosure as a means of reclaiming social belonging [33], our participants described HIV-status disclosure as being able to enable meaningful connection, but could also expose them to misunderstanding or judgment, particularly when compounded by mental health stigma.

Our study also highlighted the spatial context that shaped experiences of connection and isolation. Urban residents reported easier access to peers, community networks, and support resources, whereas rural or suburban participants experienced heightened isolation, revealing how geography and resource availability influence relational meaning and social well-being. Living in a smaller town was described as shaping access to formal supports and the availability of resonant relationships (e.g., peers). For some participants, proximity to HIV-specific organizations, clinics, and peer groups in urban centers offered opportunities for safe disclosure and authentic connection with others who shared similar lived experiences. This finding resonates with existing research showing that social isolation among older adults living with HIV can vary significantly by geography. For instance, studies have found that urban residents are more likely to access peer-based organizations and community spaces that foster belonging and resilience, while those in rural areas often experience compounded stigma and a lack of accessible, culturally competent HIV care [34,35]. Concerns about privacy and confidentiality are significant for individuals living with, or at risk of, HIV in rural communities. Many avoid testing or seeking care due to fear that someone they know, whether within their social circles or among local health staff, might recognize them or learn of their status [36]. Rural-residing individuals living with HIV also tend to underutilize mental health services due to a lack of available services compared to urban settings [37]. However, our study extends this work by revealing how geography shapes not only social access but also perceptions of shared understanding based upon the HIV resources available to them and not. Future participatory and implementation-focused research should begin to consider co-design and evaluate interventions that can mitigate spatial inequities, for example, through virtual peer networks, mobile outreach programs, and integrated HIV-mental health care models tailored to local contexts.

Virtual platforms and technology mediated forms of social engagement (e.g., video calls, online peer groups) were described by participants as a modality to maintain and create meaningful relationships when in-person engagement was limited. Earlier research has shown that older adults living with HIV are accepting of virtual technology [38]. These findings highlight technology as a tool for reducing isolation, while also raising considerations about choice, identity disclosure, and comfort in digital spaces. Our findings contrast with a study that suggested that online or virtual modes of social contact did not compensate for reduced in-person interactions during the COVID-19 pandemic and, in some cases, increased anxiety and depression, particularly among younger adults [39]. In contrast, for older adult participants living with HIV and a concurrent mental health diagnosis, digital spaces offered opportunities for selective disclosures, suggesting that virtual engagement can be not only a substitute but, in certain contexts, a preferred modality. This highlights how the value and impact of digital social engagement may differ across age groups, social contexts, and populations with shared lived experiences.

These findings point to several actionable strategies to support older adults living with HIV and concurrent mental health conditions. Clinically, healthcare providers should prioritize relational care that fosters trust, emotional safety, and selective disclosure, incorporating routine screening for social isolation and connecting patients to peer networks or mentorship opportunities. Community organizations and programming can enhance social integration by creating flexible, context-sensitive spaces (both in-person and virtual) that accommodate mobility limitations, rural or suburban residence, and privacy concerns, while facilitating reciprocal relationships and shared lived experience. At the policy level, investment in integrated HIV-mental health services, expansion of subsidized housing with peer-supportive environments, and funding for digital inclusion initiatives could reduce structural barriers to meaningful connection, promote equity across geographic settings, and address both the emotional and social dimensions of isolation identified in this study.

### Limitations

This study has several methodological limitations that limit the transferability of the findings. Participants were recruited from the Greater Toronto Area, Ontario or Ottawa, and through community organizations, which limits the findings to other geographic or cultural contexts and may over-represent individuals engaged with services. Only English-speaking participants were included, potentially excluding diverse cultural perspectives. Data relied on self-report and virtual interviews conducted once, that may have influenced depth of disclosure or non-verbal communication. The cross-sectional design limits understanding of how social isolation and relationships evolve over time, and some subgroups, including rural residents and non-binary participants, were small. Additionally, to prioritize participant comfort, specific HIV and mental health diagnoses were not collected, limiting contextualization by clinical characteristics.

Another key limitation of this study is that we did not differentiate the relative contributions of HIV-related versus mental health-related factors to participants’ experiences of social isolation and relational challenges. Our phenomenological approach focused on the lived experience of dual vulnerability as perceived by participants, capturing the integrated ways in which these factors intersect in everyday life. Future research could explore how specific diagnoses or combinations of health conditions uniquely shape social connection and isolation among older adults living with HIV.

## 5. Conclusions

This study illuminates the nuanced lived experiences of social isolation and meaningful relationships among older adults living with HIV who also have a concurrent mental health diagnosis. Participants described social isolation not merely as physical separation but as an existential experience, rooted in feeling unseen, misunderstood, or judged. Meaningful relationships were characterized by authenticity, emotional safety, and reciprocity, often emerging within peer networks of individuals with shared HIV or mental health experiences. Geography, access to community resources, and virtual platforms played a critical role in shaping social connection, highlighting both the opportunities and constraints of urban versus rural or suburban contexts. Participants emphasized the importance of selective disclosure and trust, underscoring the relational and identity-affirming dimensions of social support. Virtual engagement emerged as a flexible tool for connection, offering anonymity, choice, and access to supportive communities, while also raising considerations around privacy, identity, and comfort in digital spaces. Together, these findings suggest that interventions aiming to reduce isolation and enhance well-being for this population must extend beyond simply increasing social contacts. Clinicians, community organizations, and policymakers should create trust-based, context-sensitive spaces to support meaningful social connections, while future research should evaluate co-designed interventions and examine how age, gender, geography, and mental health shape relational experiences among older adults living with HIV.

## Figures and Tables

**Table 1:** Coding.

Code	Operational Definition
Seeking community connection	Participant actively looks for or values being around others as a way to reduce isolation or stress.
Programs as social anchors	Structured programs (e.g., yoga, HIV support groups) provide emotional or social grounding.
Social participation as coping	Engaging socially to manage mental health symptoms or avoid rumination.
Fear of isolation	Expressed worry about being alone, staying inside, or having no one to talk to.
Digital illiteracy as barrier	Difficulty using technology prevents participation in care or connection.
Desire to learn technology	Expressed motivation to gain computer or virtual literacy to connect or manage health.
Virtual connection as ‘new normal’	Recognition that healthcare and social life now rely on digital tools.
Virtual care as relational	Experience of online communication (Zoom, calls) still feels personal or meaningful.
Fear of trying technology	Anxiety about making mistakes or losing control while using technology.
Trust in provider guidance	Dependence on provider for information, referrals, or reassurance.
Provider as educator	Healthcare provider plays a teaching role, enhancing awareness and literacy.
Reliance on provider for navigation	Participant does not independently search for information; trusts provider completely.
Accessible and comprehensive care	Perception that health needs are met adequately through coordinated care.
Perceived equity in treatment	Participant reports no differential treatment or discrimination in care.
Meaningful relationship as companionship	Relationships defined by interaction, shared time, and social exchange.
Safety and nonjudgment	Trust and comfort define relational meaning more than frequency or intimacy.
Learning together	Gaining skills, coping, or literacy through collective experience.
Belonging and recognition	Feeling “seen” and included within social or program contexts.
Reciprocity as meaning	Sense of mutual exchange—talking, laughing, sharing.
Avoidance of stress and overthinking	Actively limiting worry or introspection to protect mental health.
Programs as mental health maintenance	Structured engagement helps manage stress and provides routine.
Spiritual or emotional resilience	Positive acceptance or focus on gratitude.
Physical engagement as wellness	Activities like yoga integrate body and mind care.
Sense of gratitude for system support	Expressed appreciation for accessible care or medication coverage.
Financial constraint as limiting participation	Economic limitations affect ability to access certain health services.
Government or community reliance	Acknowledgement of public or community systems as lifelines.
Living alone	References to solitary living, associated with isolation or independence.
Being-with (Mitsein)	Expressed need to be with others as constitutive of well-being.
Being-in-the-world-with-technology	Reorientation of self toward virtual forms of presence and interaction.
Being-cared-for	Ontological security found in dependable healthcare relationships.
Being-toward-learning	Openness to growth and adaptation, even in later life.

**Table 2:** Participant Demographics (*n* = 30/33).

Age	50–54	5
	55–59	9
	60–64	6
	65–69	5
	70–74	1
	75–79	2
	80+	2
Living setting	Urban	24
	Suburban	2
	Rural	4
Country of Birth	Canada	8
	Zimbabwe	1
	Guyana	1
	Israel	1
	Malaysia	2
	United Kingdom	1
	South Africa	1
	Trinidad and Tobago	1
	Other, prefer not to answer	14
If not Canada, how long have you been in Canada?	0–5 years	4
	6–10 years	3
	10+ years	9
First language	English	21
	Other	9
Racial and ethnic background	South Asian	5
	Black African	4
	Caribbean	2
	Indigenous	2
	European White	6
	North American White	10
	Mixed	1
Gender	Man	19
	Woman	10
	Non-binary	1
Sexual orientation	Heterosexual	12
	Gay	14
	Pansexual	1
	Queer	1
	Asexual	2
Household income before taxes	$0–$29,999	17
	$30,000–$59,000	4
	$60,000–$89,999	5
	$90,000–$119,999	3
	$150,000 or more	1
Number of people in household	0 (precariously housed)	1
	1	19
	2	10
Highest level of education completed	Less than high schoo	4
	High school or equivalent	6
	Some college or university	8
	Degree or diploma from college or university	6
	Graduate degree	6
Disabilities *	Visual impairment	8
	Hearing impairment	3
	Mobility impairment	5
	Mental health disorder	8
	Other	1
	No disabilities	12

* some participants selected more than one answer.

## Data Availability

The data that support the findings of this study are available from the corresponding author upon reasonable request, respecting confidentiality and ethical considerations.
